# Differences of serum glucose and lipid metabolism and immune parameters and blood metabolomics regarding the transition cows in the antepartum and postpartum period

**DOI:** 10.3389/fvets.2024.1347585

**Published:** 2024-02-02

**Authors:** Xinya Zhao, Yuxin Wang, Luyao Wang, Shouqiang Sun, Chaoyue Li, Xuewei Zhang, Long Chen, Yujia Tian

**Affiliations:** ^1^Tianjin Key Laboratory of Agricultural Animal Breeding and Healthy Husbandry, College of Animal Science and Veterinary Medicine, Tianjin Agricultural University, Tianjin, China; ^2^Tianjin Jialihe Animal Husbandry Group Co., Ltd., Tianjin, China; ^3^Beijing Dongfang Lianming Technology Development Co., Ltd., Beijing, China

**Keywords:** transition period, metabolomics, glucose metabolism, lipid metabolism, metabolic pathway

## Abstract

This study aims to investigate differences in metabolism regarding the transition cows. Eight cows were selected for the test. Serum was collected on antepartum days 14th (ap14) and 7th (ap7) and postpartum days 1st (pp1), 7th (pp7), and 14th (pp14) to detect biochemical parameters. The experiment screened out differential metabolites in the antepartum (ap) and postpartum (pp) periods and combined with metabolic pathway analysis to study the relationship and role between metabolites and metabolic abnormalities. Results: (1) The glucose (Glu) levels in ap7 were significantly higher than the other groups (*p* < 0.01). The insulin (Ins) levels of ap7 were significantly higher than pp7 (*p* = 0.028) and pp14 (*p* < 0.01), and pp1 was also significantly higher than pp14 (*p* = 0.016). The insulin resistance (HOMA-IR) levels of ap7 were significantly higher than ap14, pp7, and pp14 (*p* < 0.01). The cholestenone (CHO) levels of ap14 and pp14 were significantly higher than pp1 (*p* < 0.01). The CHO levels of pp14 were significantly higher than pp7 (*p* < 0.01). The high density lipoprotein cholesterol (DHDL) levels of pp1 were significantly lower than ap14 (*p* = 0.04), pp7 (*p* < 0.01), and pp14 (*p* < 0.01), and pp14 was also significantly higher than ap14 and ap7 (*p* < 0.01). (2) The interferon-gamma (IFN-γ) and tumor necrosis factor α (TNF-α) levels of ap7 were significantly higher than pp1 and pp7 (*p* < 0.01); the immunoglobulin A (IgA) levels of pp1 were significantly higher than ap7 and pp7 (*p* < 0.01); the interleukin-4 (IL-4) levels of pp7 were significantly higher than ap7 and pp1 (*p* < 0.01), the interleukin-6 (IL-6) levels of ap7 and pp1 were significantly higher than pp7 (*p* < 0.01). (3) Metabolomics identified differential metabolites mainly involved in metabolic pathways, such as tryptophan metabolism, alpha-linolenic acid metabolism, tyrosine metabolism, and lysine degradation. The main relevant metabolism was concentrated in lipid and lipid-like molecules, organic heterocyclic compounds, organic acids, and their derivatives. The results displayed the metabolic changes in the transition period, which laid a foundation for further exploring the mechanism of metabolic abnormalities in dairy cows in the transition period.

## Introduction

During the transition period, cows have to go through complicated physiological processes, such as pregnancy, delivery, and lactation (1). These physiological processes will change the nutritional needs and metabolism of the body. These changes are caused by the increasing nutritional and energy needs at the beginning of milk production, which will result in a negative balance state and even cause metabolic abnormalities, such as insulin resistance and various metabolic diseases (2). The nutritional requirements of peripartum cows, such as carbohydrates, fat, and protein, vary with physiological changes from calving to lactation (3). Large amount of glucose is lost to the breast during pregnancy and lactation, initiating the use of carbohydrate resources (4). Due to the limited supply of glucose in feed energy intake, cows lack carbohydrate intake for a long time, and lactation consumes a large amount of energy, resulting in lower blood glucose. To satisfy energy requirement, the body prioritizes mobilizing fat storage for milk production (5). Triglycerides in adipose tissues are subjected to lipolysis, releasing large amount of non-esterified fatty acids into the blood circulation and esterifying in the liver. However, when the amount of non-esterified fatty acids exceeds the esterification ability of the liver, ketone bodies are formed, resulting in an increase in β-hydroxybutyric acid and the occurrence of ketosis (6). Currently, to prevent and improve the NEB status, nutritional regulation is adopted, for instance, probiotics, prebiotics, dietary lipids, and Chinese medicine compounds, which may also reduce the risk of postpartum disease (7–9). The physiological mechanisms related to the transitional period in dairy cows are not fully understood, and it is unclear whether mutual synergy can better prevent and treat transitional metabolic diseases. Only limited measures can treat metabolic diseases in transition cows, and the investigators try to use metabolomics to address this problem. Metabonomics is helpful to further understand the physiological mechanism of dairy cows in transition period, so as to identify different blood metabolites before and after calving. Thus, providing information is helpful to treat and prevent metabolic diseases of dairy cows in transition period. This method is widely used in the treatment of subclinical mastitis, placenta retention, and ketosis in cows (10–13). Previous studies have assessed the dynamic changes in biological amines, acylcarnitines, glycerophospholipids, and sphingolipids, based on targeted metabolomics. Blood samples at 28 days postpartum showed some increases in AA and sphingomyelin when compared with 7 days postpartum (14). Only the longitudinal changes in the blood metabolome were analyzed to identify new biomarkers. However, few studies clearly indicate that metabolic pathway changes in the whole transition period.

Untargeted metabolomics can detect metabolite differences between the control and test groups (15, 16). Although studies of metabolic diseases in cows have been reported previously, there are still no clearly effective prevention and treatment options in production.

We hypothesized that cows may cause changes in metabolites before and after delivery and may reveal metabolite biomarkers that reflect cows before and after delivery. In this study, biochemical and immune indexes of serum samples from transition cows were determined, and plasma samples from transition cows were analyzed by untargeted metabolomics methods. Moreover, differential metabolites and major metabolic pathways were identified. Meanwhile, biochemical and immune indexes were correlated with differential metabolites. These trials may aid in the prevention and treatment of metabolic abnormalities in cattle and explain the underlying metabolic mechanisms.

## Materials and methods

### Serum sample collection and biochemical measurements

The present study was carried out in Tianjin Haorui Feng Animal Husbandry Co., Ltd. (Tianjin, China) during February 2022. Twenty transitional cows were randomly selected and eight healthy Holstein cows with similar body condition (3.25 ± 0.5, 5-point scale for body condition score), weight (570 ± 50 kg), and similar pregnancy days were finally selected for testing. The TMR formulas for the pre-partum period and the post-partum period were formulated following the NRC requirements (2001). The cows were fed twice, in the morning and evening, with free water access. Table 1 presents the composition of TMR and the nutritional values. Blood samples were collected from the caudal vein of cows antepartum 14th and 7th days and postpartum 1st, 7th, and 14th days in the morning. Two 10 mL vacuum blood collection tubes and one 10 mL vacuum blood collection tube containing Ethylenediaminetetraacetic acid (EDTA) were used for collection. The whole blood in EDTA tube was directly subpackaged in 2 mL cryovials, and two vacuum blood collection tubes were centrifuged at 3,500 rpm/min for 10 min. The upper serum was collected and subpackaged in 2 mL cryovials, all of which were stored at −80°C refrigerator. Blood samples collected were grouped by time, and serum at antepartum 14th and 7th days and postpartum 1st, 7th, and 14th days was grouped as ap14 and ap7 and pp1, pp7, and pp14, respectively. Moreover, the whole blood in EDTA tube at ap14, ap7, pp1, pp7, and pp14 day was grouped as ap and pp, respectively.

**Table 1:** Ingredient composition of diets during the peripartum period.

Items	Prepartum	Postpartum
**Ingredient (%)**		
Oat hay	25	7
Alfalfa haylage		11
Corn silage	35	29
Cotton seed		4.5
Corn grain ground fine	12	8
Soybean meal	10	5.5
Low erucic acid and low glucoside rapeseed meal	16	15
Haylage		10
Steam corn flakes		4.5
Antifungal agent		0.2
Rumen by pass fats		0.8
Sodium bicarbonate		1.5
Mineral premix	2^1^	3^2^
**Chemical analysis** ^3^		
Net energy for lactation^3^, Mcal/kg	1.31	1.74
Dry matter (%)	51.04	53.96
Crude ash (%)	6.21	7.81
Crude protein (%)	18.66	14.83
Crude fiber (%)	1.98	4.28
Neutral detergent fiber (%)	38.44	22.09
Acid detergent fiber (%)	22.16	14.46

^1^Each kilogram of prepartum premix contains vitamin A 32,000 IU, vitamin D3 7,000 IU, dl-a-tocopherol acetate 2,500 mg, and selenium 1.5 mg.

^2^Each kilogram of postpartum premix contains vitamin A 35,000 IU, vitamin D3 10,000 IU, dl-a-tocopherol acetate 2,600 mg, copper 340 mg, manganese 1,500 mg, and zinc 1,500 mg.

^3^Net energy for lactation is the calculated value; the others are the measured values.

### Biochemical and immune measurements

The estimated due date of each cow was estimated, and the glucose levels of the cows were measured using a glucose meter (OGM-161, Harold Beijing Technology Co., Ltd., China) at ap14, ap7, pp1, pp7, and pp14, respectively. In total, 2 mL of frozen storage tubes containing serum was unfrozen.

Measurement method of biochemical indexes: insulin, glycosylated hemoglobin (HbA1c), glucagon (GC), and cholinesterase (AChE) contents of each cow at ap14, ap7, pp1, pp7, and pp14 were measured using enzyme-linked immunosorbent assay (ELISA) kit (Shanghai Jianglai Biotechnology Co., Ltd., China) and Bio-Rad iMark. HOMA-IR (HOMA-IR = Glu (mmol/L) × Ins (mIU/L)/22.5) index was calculated by the steady-state model evaluation method (17, 18). CHO by CHOD-PAP method and triglyceride (TG) by GPO-PAP method, low density lipoprotein cholesterol (DLDL) by direct method-surfactant removal method, and DHDL by direct method-catalase removal method. The kits were purchased from Zhongsheng Beikong Biotechnology Co., Ltd. The levels of CHO, TG, DLDL, and DHDL were analyzed using a fully automated biochemical analyzer (GLAMOUR 3000, Molecular Devices Co., Ltd., America) at ap14, ap7, pp1, pp7, and pp14 in each cow.

Measurement method of immune indexes: interferon-gamma (IFN-γ), immunoglobulin A (IgA), immunoglobulin G (IgG), immunoglobulin M (IgM), interleukin-1β (IL-1β), interleukin-4 (IL-4), interleukin-6 (IL-6), and tumor necrosis factor α (TNF-α) contents of each cow at ap7, pp1, and pp7 were measured using enzyme-linked immunosorbent assay (ELISA) kit (Shanghai Jianglai Biotechnology Co., Ltd., China) and Bio-Rad iMark.

### Untargeted liquid chromatography-MS metabolomic sample measurements

A thawed serum sample (100 μL) was added to a 1.5 mL centrifuge tube, and 400 μL of methanol: acetonitrile (vol/vol = 1:1) was added for extraction. The samples were mixed by vortexing for 30 s and sonicating at 40 kHz and 5°C for 30 min. The samples were allowed to stand at −20°C for 30 min. The supernatants were then centrifuged for 15 min at 13,000 × g and 4°C and dried with nitrogen. To the sample, 120 μL of reconstituted solution (acetonitrile: water =1:1) was added for reconstitution and vortexed again for 30 s. After ultrasonic extraction for 5 min at 40 kHz and 5°C and centrifugation for 10 min at 13,000 × g and 4°C, the supernatants were transferred to autosampled vials and analyzed on the computer. All samples were mixed with 20 μL of supernatant per sample as quality control (QC) samples, and one QC sample was inserted in every 5–15 analysis samples to examine the stability of the overall assay. After the completion of sample pretreatment, the samples were subjected to liquid chromatography-tandem mass spectrometry analysis.

Each sample was analyzed on an Ultra Performance Liquid Chromatography Tandem Fourier Transform Mass Spectrometry (UHPLC-Q Exactive HF-X) system. The system column is ACQUITY UPLC HSS T3 (100 mm × 2.1 mm I.D., 1.8 μm). The column temperature was 40°C. The mobile phase consisted of A (95% water + 5% acetonitrile + 0.1% formic acid) and B (47.5% acetonitrile + 47.5% isopropanol + 5% water + 0.1% formic acid). The gradient elution procedure is presented in Supplementary Table S1, and the injection volume was 3 μL. Samples were ionized by electrospray and collected in positive and negative ion scanning modes (Supplementary Tables S1, S2).

### Serum biochemical and immune data processing and statistical analyses

The data of biochemical indexes and immune indexes of cows were analyzed by one-way ANOVA and multiple comparisons with Tukey’ s honestly significant difference as *post hoc* test procedure using SPSS software (version 25.0, IBM SPSS). Differences were declared significant at *p* < 0.05 and were declared extremely significant at *p* < 0.01.

### Metabolomics data processing and statistical analyses

The raw data were imported into the metabolomics processing software ProgenesisQI (Waters Corporation, Milford, United States) for baseline filtering, peak identification, retention time correction, and peak alignment and finally obtained a data matrix of retention time, mass-charge ratio, and peak intensity. Subsequently, the software was used to identify the feature peak search library and matched the MS and MS/MS mass spectral information with metabolic public database HMDB,^1^ Metlin,^2^ and Majorbio self-built library. MS mass error was set to less than 10 ppm while metabolites were identified based on second-order MS matching scores. After searching, the matrix data were uploaded to Meiji Biological Cloud Platform^3^ for data analysis. Less than 20% of the ion peaks were removed, and the minimum was used to fill the vacancy values and sum normalized, thus reducing sample and instrument errors. The Variables with relative deviation ≤30% (generally, variables with RSD > 30% fluctuate too much during the experiment) were excluded. Then log10 was used to transform the data, so as to improve the normal distribution of the data structure and reduce the analysis error. The R software package ropls (version 1.6.2) performs orthogonal least squares-discriminant analysis (OPLS-DA) to evaluate the stability of the model. OPLS-DA can eliminate the noise information irrelevant to the classification and can also obtain the relevant metabolite information that leads to significant differences between the two groups. To add a positive exchange calculation over partial least squares-discriminant analysis (PLS-DA), OPLS-DA was used. It filters out the signals irrelevant to the model classification and has interpretation ability. The OPLS-DA model was validated based on Y (R2Y) modeling ability and model (Q2) with 200 iterations and the OPLS-DA displacement test. When the R2Y and Q2 indicators are closer to 1, the more stable and reliable the model is. The Q2 regression line intercept is less than 0, and the model is not overfit. Moreover, Q2 > 0.5 indicates better predictive power of the model (19).

Significant different metabolites were screened based on the variable weight values (VIP) and student’s *t*-test *p*-values obtained from the OPLS-DA model. Metabolites with VIP >1 and *p* < 0.05 were considered as differential metabolites. Characteristic peak search library identification was performed, and MS information was matched with the metabolic database. MS mass error was set to less than 10 ppm while metabolites were identified based on the secondary MS mating score. The main databases are http://www.hmdb.ca/, https://metlin.scripps.edu/, and other mainstream public databases and self-built databases. In addition, pathways involving differential metabolites were obtained through the metabolic pathway annotation of the KEGG database.^4^ Clustering and pathway analysis data were processed and analyzed using the Python software package scipy.stats, to obtain the biological pathway, which was most relevant to experimental processing by Fisher’s exact test.

## Results

### Univariate analysis of biochemical indicators

Tables 2, 3 present the results of glucose and lipid metabolism at different times during the transition period. In Table 2, the Glu levels in ap7 were significantly higher than the other time points (*p* < 0.01); the Ins levels of ap7 were significantly higher than pp7 (*p* = 0.028) and pp14 (*p* < 0.01), and pp1 was also significantly higher than pp14 (*p* = 0.016); HOMA-IR levels of ap7 were significantly higher than ap14, pp7, and pp14 (*p* < 0.01). In Table 3, the CHO levels of ap14 and pp14 were significantly higher than pp1 (*p* < 0.01), and the CHO levels of pp14 were significantly higher than pp7 (*p* < 0.01); DHDL levels of pp1 were significantly lower than ap14 (*p* = 0.041), pp7 (*p* < 0.01), and pp14 (*p* < 0.01), and pp14 was also significantly higher than ap14 and ap7 (*p* < 0.01).

**Table 2:** Index related to glucose metabolism at different time in the transition period (*n* = 8).

Index	Time					SEM^4^	*p*-value
	ap14^3^	ap7	pp1	pp7	pp14		
Glu	2.56^b2^	4.78^a^	3.19^b^	2.83^b^	2.99^b^	0.433	<0.01
(mmol/L)							
Ins	11.22^abc^	20.80^a^	17.07^ab^	10.06^bc^	5.59^c^	3.445	<0.01
(mIU/L)							
HOMA-IR^1^	1.28^b^	4.52^a^	2.63^ab^	1.21^b^	0.74^b^	0.697	<0.01
HbA1c	6.81	8.41	8.53	7.27	6.68	1.236	0.418
(ng/mL)							
GC	126.44	183.73	167.18	128.16	81.77	39.953	0.118
(pg/mL)							

^1^HOMA-IR (HOMA-IR = Glu (mmol/L) × Ins (mIU/L)/22.5) index was calculated by the steady-state model evaluation method, which is the coefficient of insulin resistance.

^2^a–c Means within a row with different letters differed significantly (*p* < 0.05). Same as below.

^3^The ap is prenatal and the pp is postpartum. Same as below.

^4^SEM is representing the differences between the groups obtained from the multiple comparison method. Same as below.

**Table 3:** Index related to lipid metabolism at different time in the transition period (*n* = 8).

Index	Time					SEM	*p*-value
	ap14	ap7	pp1	pp7	pp14		
AChE	62.09	66.20	57.21	47.53	41.15	15.645	0.498
(nmol/L)							
TG	0.50	0.48	1.18	0.34	0.47	0.433	0.377
(mmol/L)							
CHO	2.17^ab^	2^abc^	1.45^c^	1.78^bc^	2.54^a^	0.206	<0.01
(mmol/L)							
DLDL	0.22	0.2	0.13	0.22	0.16	0.059	0.489
(mmol/L)							
DHDL	1.21^b^	1.13^bc^	0.85^c^	1.36^ab^	1.67^a^	0.121	<0.01
(mmol/L)							

### Univariate analysis of immune indicators

Table 4 present the results of immune performance at different times during the transition period. The IFN-γ and TNF-α levels of ap7 were significantly higher than pp1 and pp7 (*p* < 0.01); the IgA levels of pp1 were significantly higher than ap7 and pp7 (*p* < 0.01); the IL-4 levels of pp7 were significantly higher than ap7 and pp1 (*p* < 0.01), the IL-6 levels of ap7 and pp1 were significantly higher than pp7 (*p* < 0.01).

**Table 4:** Index related to immune performance at different time in the transition period (*n* = 8).

Index	Time			SEM	*p*-value
	ap7	pp1	pp7		
IFN-γ	607.43^a^	310.64^b^	332.25^b^	29.933	<0.01
(mIU/L)					
IgA	315.75^b^	906.06^a^	282.31^b^	61.575	<0.01
(ng/mL)					
IgG	2.80	2.61	2.14	0.159	0.228
(pg/mL)					
IgM	550.25	583.06	558.06	13.833	0.620
(mmol/L)					
IL-1β	237.73	260.17	190.01	12.900	0.070
(mIU/L)					
IL-4	2.64^b^	1.80^b^	11.42^a^	0.925	<0.01
(ng/mL)					
IL-6	86.97^a^	81.43^a^	58.42^b^	4.192	<0.01
(pg/mL)					
TNF-α	18.75^a^	15.57^b^	14.92^b^	0.519	<0.01
(pg/mL)					

### Analysis of metabolite profiles of plasma

**Figure 1:**
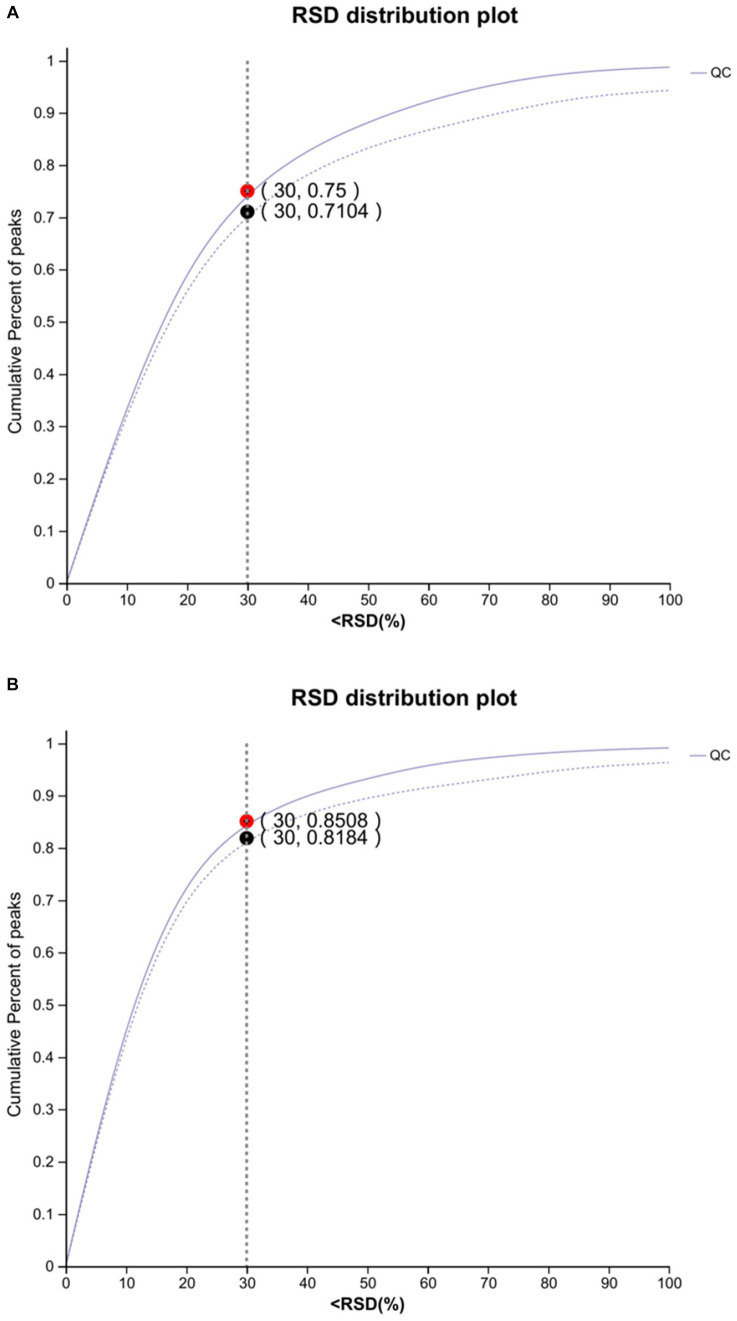
Plot of anion **(A)** and cation **(B)** assessment of QC samples (*n* = 8). The abscissa is the RSD (%) value, the standard deviation/mean, and the ordinate is the cumulative proportion of the ion peaks. (The dotted line indicates before pretreatment and the solid line indicates after pretreatment).

The results of the RSD represent the degree of dispersion of the data. A lower RSD represents a data point closer to the mean. The standard deviation of the data set is less than 30% of the mean, confirming the consistency and stability of the data. For the overall data, if the QC sample assessment map is RSD <0.3 and the cumulative proportion of peaks is >70%, the overall data are qualified (Figure 1).

**Figure 2:**
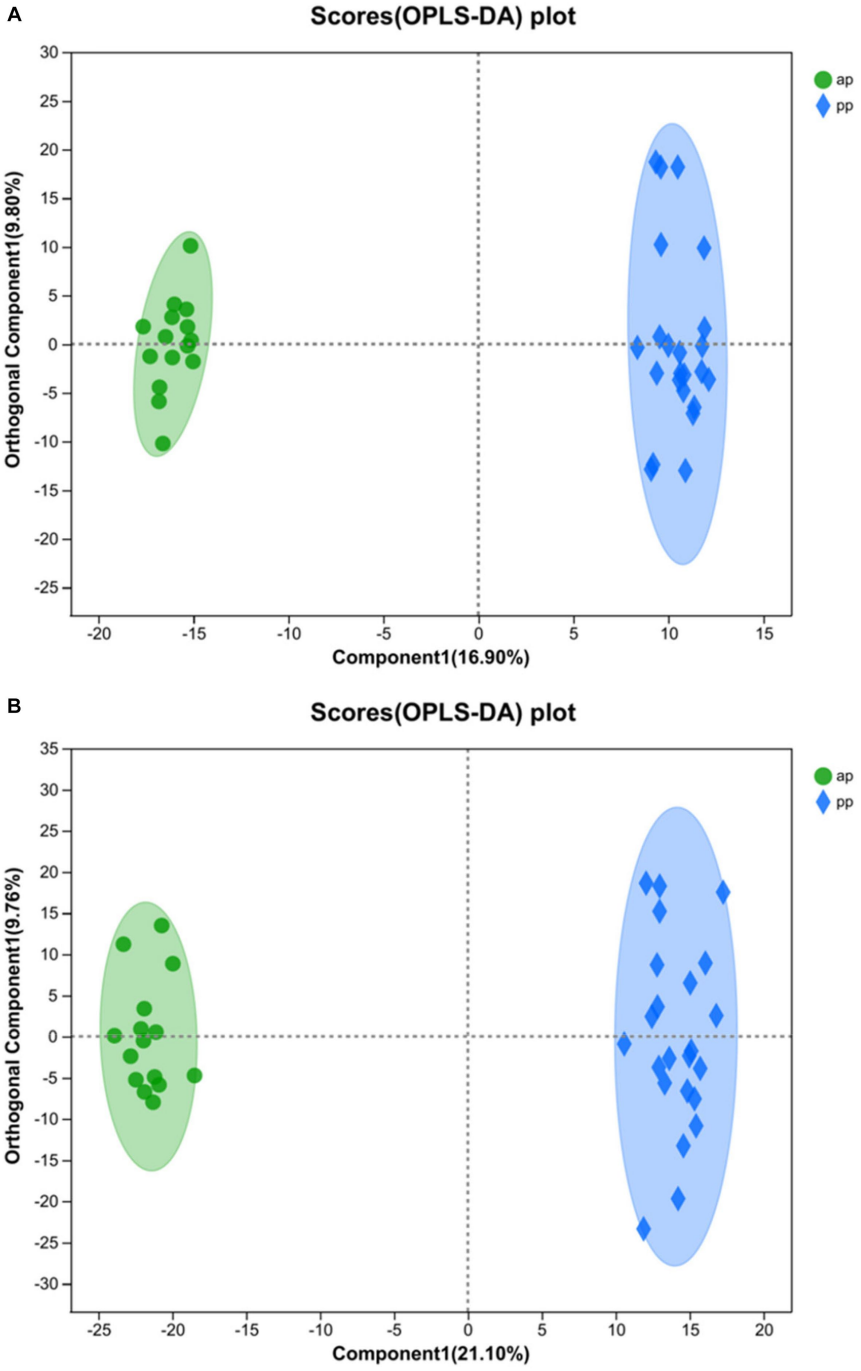
OPLS-DA score chart of ap and pp in serum samples for cation **(A)** and anion **(B)** pattern analysis (*n* = 8). Comp1 first predicted principal component interpretation degree, orthogonal Comp1 first orthogonal component interpretation degree. And ap, antenatal sampling time; pp, postnatal sampling time. Same as below.

**Figure 3:**
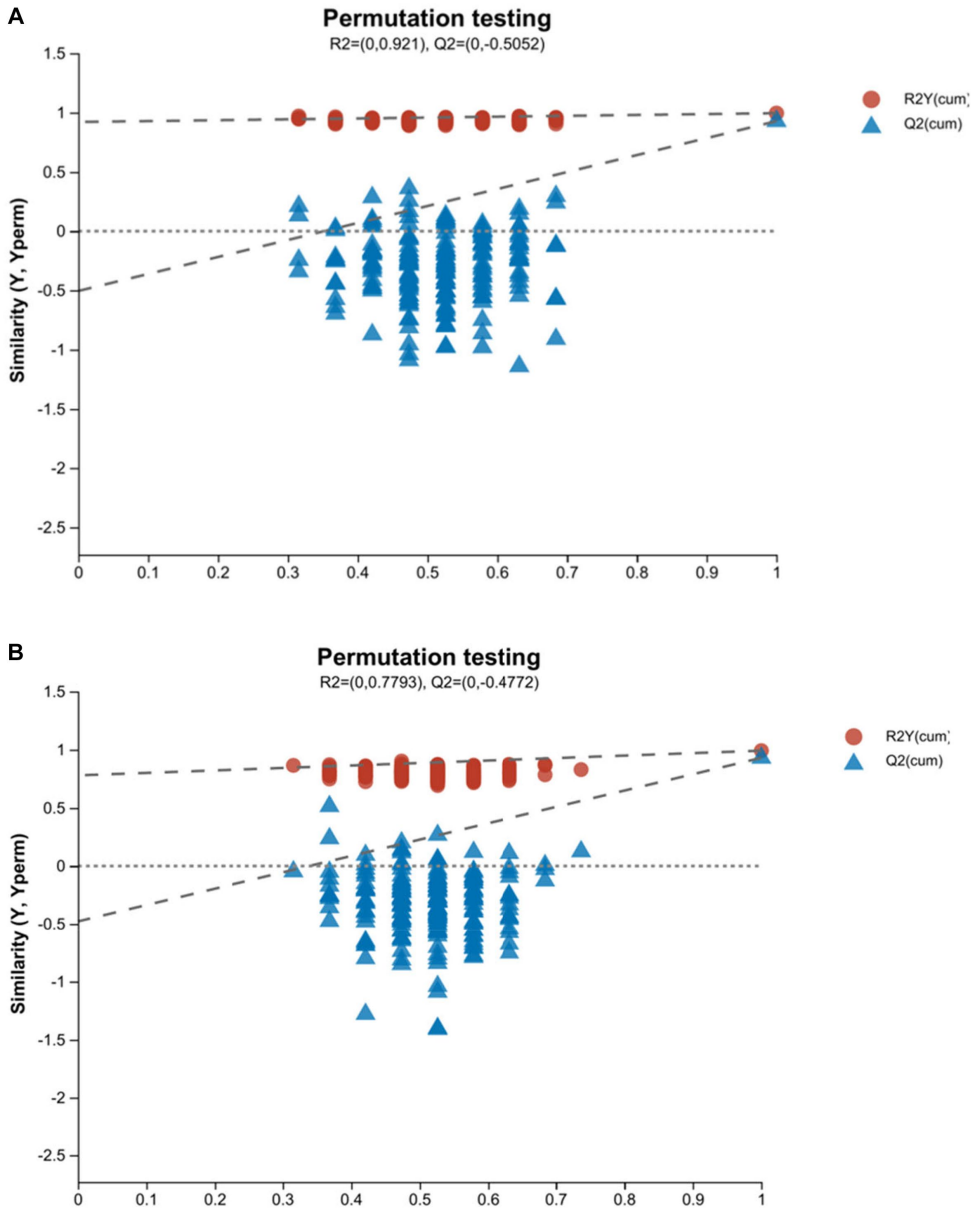
OPLS-DA model validation plots of ap and pp in serum samples for cation **(A)** and anion **(B)** pattern analysis (*n* = 8). In this model, R2 is the model interpretation rate, and Q2 is the prediction ability of the model. The abscissa represents the displacement retention degree of the displacement test (the proportion consistent with the order of the *Y* variable in the original model. The point where the displacement retention degree is equal to 1 is the R2 and Q2 values of the original model). The ordinate represents the values of R2 and Q2. The blue dot represents the R2 value obtained by the displacement test. The red square point represents the Q2 value obtained by the displacement test. The two dotted lines, respectively, represent the regression lines of R2 and Q2.

R2Y (*cum*) and Q2 (*cum*) are used to express the evaluation on the success of OPLS-DA model establishment. The model reliability is expressed as R2Y& Q2 >0.5 (19). The closer to 1, the stronger the reliability is. In the positive ion mode of the OPLS-DA score plot, R2Y = 0.995 and Q2 = 0.928, whereas in the negative ion mode, R2Y = 0.992 and Q2 = 0.93. Both R2Y and Q2 values were greater than 0.5, indicating that the model was stable and reliable (Figures 2A,B). The Q2 intercept values were less than 0.05 (20), indicating that there was no overfitting (Figures 3A,B). The above data indicated that the OPLS-DA model in the ap group and pp group had strong prediction ability, the model was successfully established, and the experimental data were reliable.

### Analysis of differential metabolite

**Figure 4:**
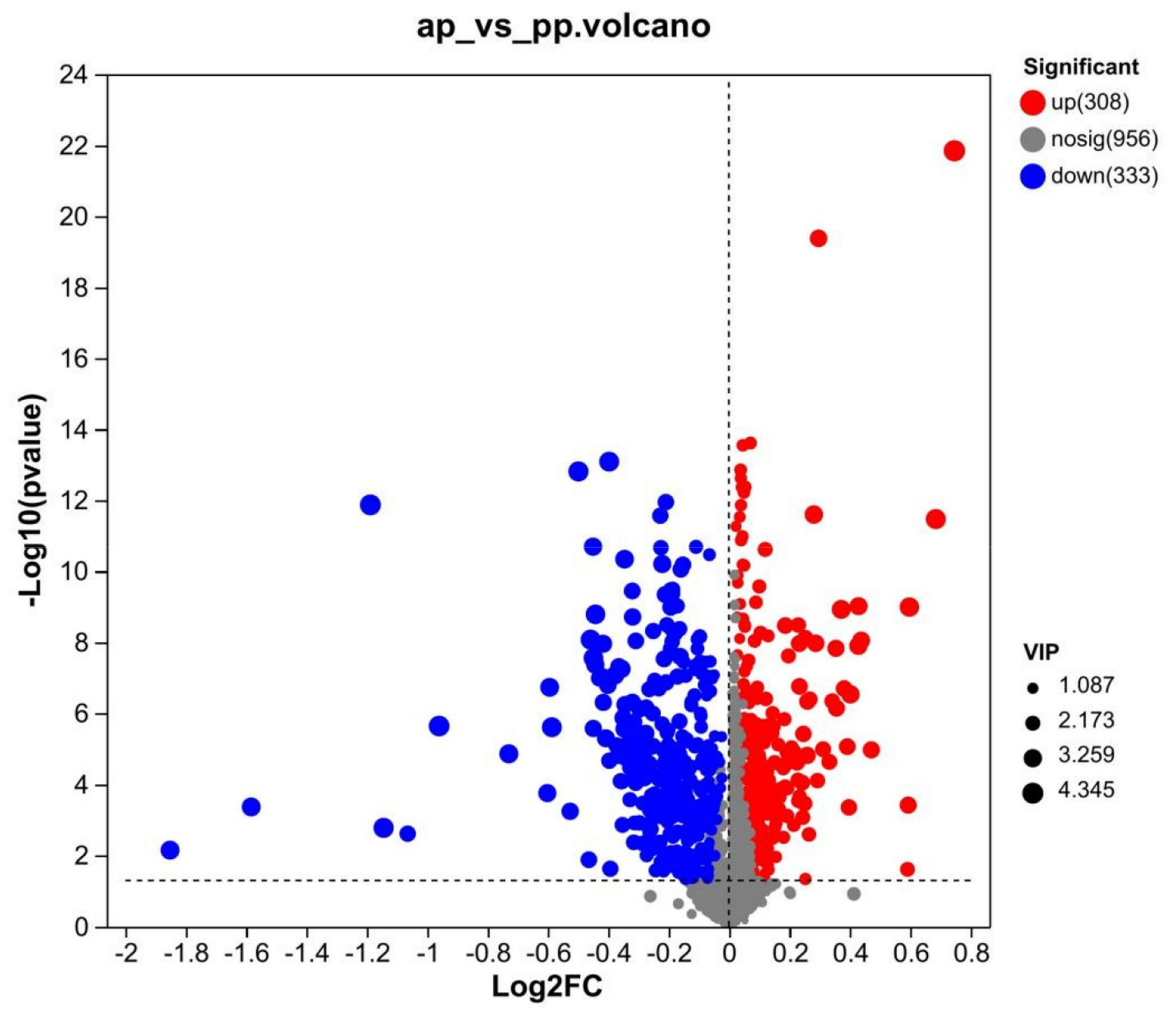
Volcano map of differential metabolite screening for groups ap and pp (*n* = 8). Each point in the volcanic map represents a metabolite. The abscissa represents the multiple change (the base-2 log) of the group against each substance, and the ordinate represents the *p*-value (the base-10 log) of student’s *t*-test. The scatter colors represented the final screening results, with significantly different upregulated metabolites indicated in red, significantly different downregulated metabolites indicated in green, and non-significantly different metabolites indicated in grey.

**Figure 5:**
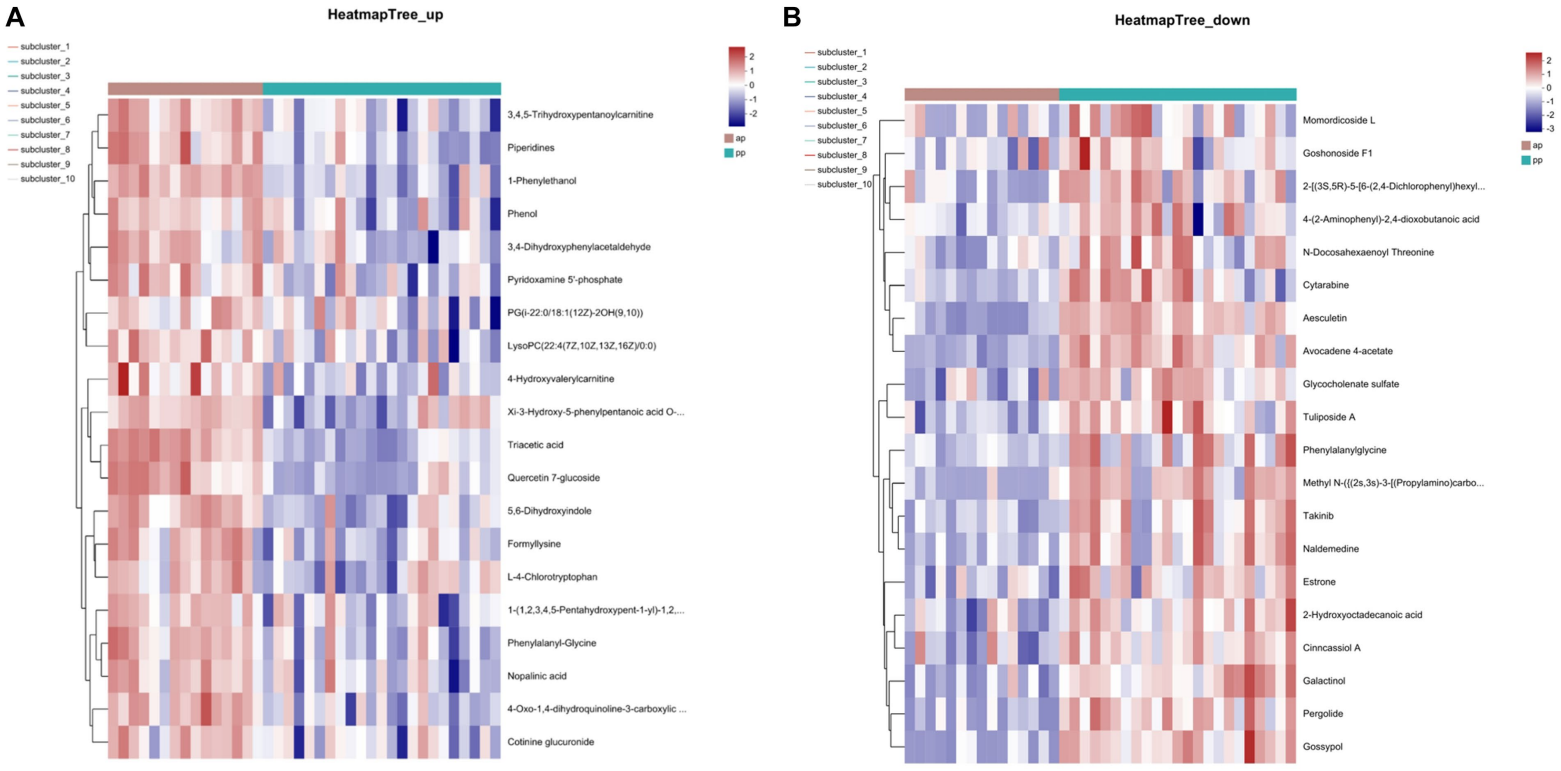
Heatmap of the top 20 differential metabolites identified in abundances in serum samples for cation **(A)** and anion **(B)** pattern analysis (*n* = 8).

A total of 641 differential metabolites were identified, of which, 308 metabolites were upregulated and 333 were downregulated in prenatal compared with postpartum (Figure 4). The cluster heatmap showed that similar metabolites were located in adjacent locations, and the dendrogram showed that the samples from the ap and pp groups can be separated (Figures 5A,B).

**Figure 6:**
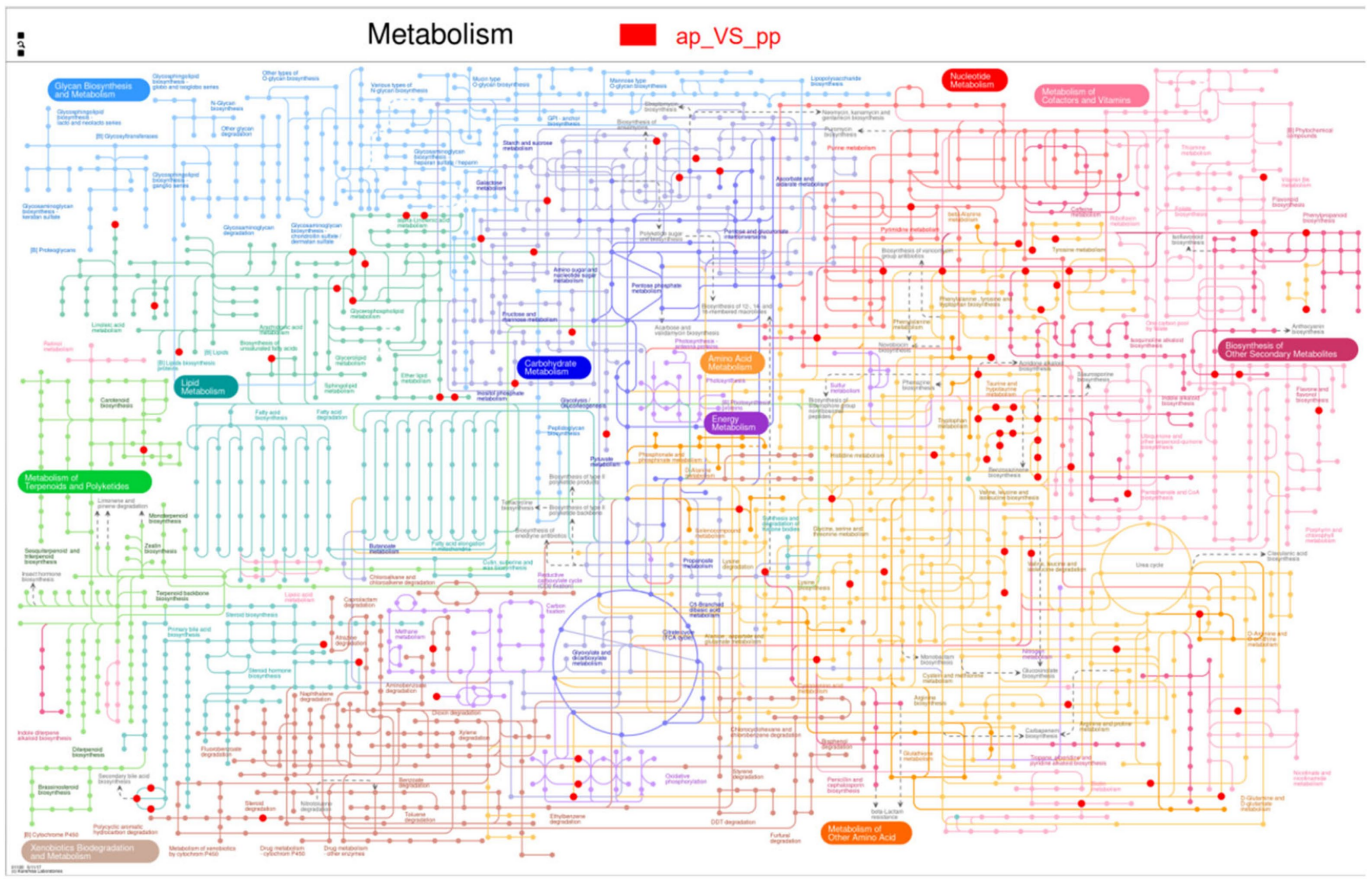
Differential metabolites are annotated to the global overview map (*n* = 8). The pictures represent the pathways annotated by the metabolic set, and one or two metabolic sets can be analyzed; when two metabolic sets are analyzed, different colors represent the pathways annotated by the metabolites in different metabolic sets, and blue represents the pathways jointly annotated by the two metabolic sets. Red dots are the metabolites.

**Figure 7:**
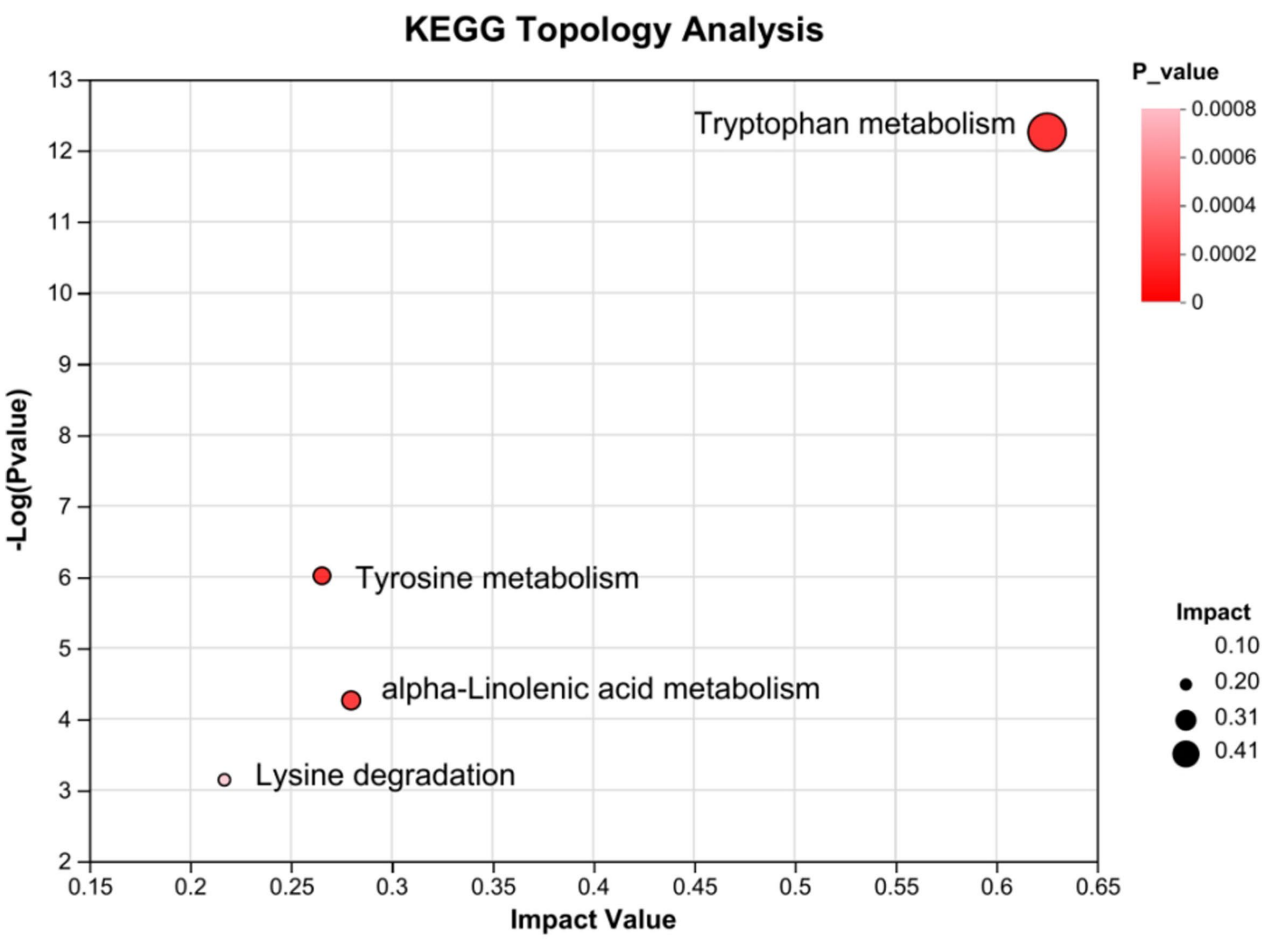
KEGG pathway enrichment bubble map by topology analysis (*n* = 8). Each bubble in the figure represents a KEGG pathway; the horizontal axis indicates the relative importance of the metabolites in the pathway impact value; the vertical axis indicates the significance of the metabolites in the pathway-log10 (*p*-value); the bubble size represents the impact value; the larger the bubble, the greater the importance of the pathway.

Subsequently, the differential metabolites in the Kyoto Encyclopedia of Genes and Genomes pathway database were queried. Metabolic pathways were visualized using iPath 3.0.^5^ The nodes in the global overview map represent different compounds, and the boundaries represent different enzymatic reactions (Figure 6). The identified global metabolic pathways were mainly involved in AA metabolism, lipid metabolism, and carbohydrate metabolism. To further screen the pathways, metabolic pathway analysis using metabolic analysis software were performed, and the key pathways with the highest correlation with metabolite differences were found. In total, four major metabolic pathways were affected >0.2, *p* < 0.05. They were tryptophan metabolism, alpha-linolenic acid metabolism, tyrosine metabolism, and lysine degradation (Figure 7).

### Association analysis between metabolites and biochemical indicators

**Figure 8:**
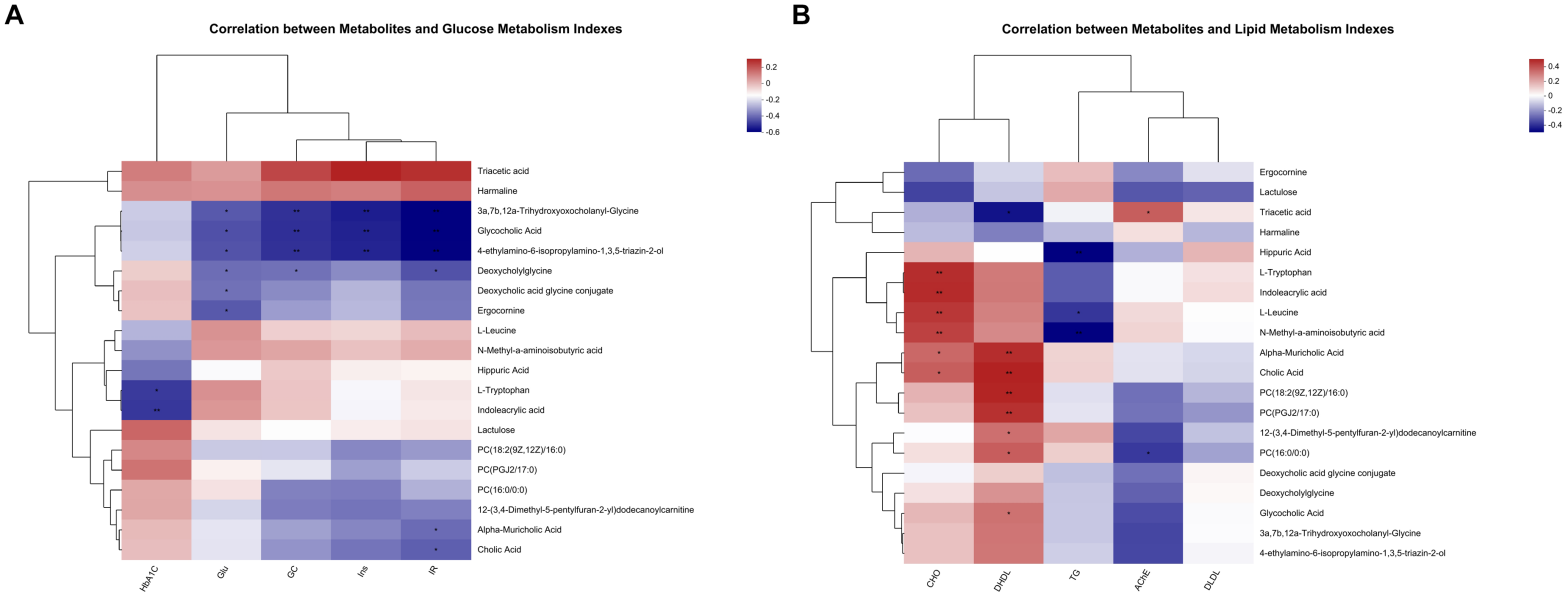
**(A,B)** Shows how the correlation of metabolites with glucose index and the correlation of metabolites with lipid index, respectively (*n* = 8).

Metabolites with glucose metabolism index and lipid metabolism index were correlated, respectively. The top 20 abundance metabolites were selected, and the heatmap of their top 50 abundance correlation features is presented in Figure 8. In Figure 8A, Ins levels were compared with 3a, 7b, 12a-trihydroxyoxocholanyl-glycine, glycocholic acid, 4-ethylamino-6-isopropylamino-1, and 3,5-triazin-2-ol, which showed a significant negative correlation. Moreover, Glu level was significantly negatively associated with 3a, 7b, 12a-trihydroxyoxocholanyl-glycine, glycocholic acid, 4-ethylamino-6-isopropylamino-1, 3,5-triazin-2-ol, deoxycholylglycine, deoxycholic acid glycine conjugate, and ergocornine. HOMA-IR showed significant negative correlation with 3a, 7b, 12a-trihydroxyoxocholanyl-glycine, glycocholic acid, 4-ethylamino-6-isopropylamino-1, 3,5-triazin-2-ol, deoxycholylglycine, alpha-muricholic acid, and cholic Acid. HbA1C level showed significant negative correlation with L-tryptophan and indoleacrylic acid. The GC levels showed a significant negative correlation with 3a, 7b, 12a-trihydroxyoxocholanyl-glycine, glycocholic acid, 4-ethylamino-6-isopropylamino-1, 3,5-triazin-2-ol, and deoxycholylglycine.

As shown in Figure 8B, AChE levels showed a significant negative correlation with PC (16:0/0:0) and a significant positive correlation with triacetic acid. The TG levels showed a significant negative correlation with hippuric acid, L-leucine, and N-methyl-a-aminoisobutyric acid and significant positive correlation of CHO levels with L-tryptophan, indoleacrylic acid, L-leucine, N-methyl-a-aminoisobutyric acid, alpha-muricholic acid, and cholic Acid. DHDL levels showed significant positive correlation with alpha-muricholic acid, cholic acid, PC (18:2 (9Z, 12Z)/16:0), PC (PGJ 2/17:0), 12-(3,4-dimethyl-5-pentylfuran-2-yl) dodecanoylcarnitine, PC (16:0/0:0), and glycocholic acid and significant negative correlation with triacetic acid.

### Association analysis between metabolites and immune indicators

**Figure 9:**
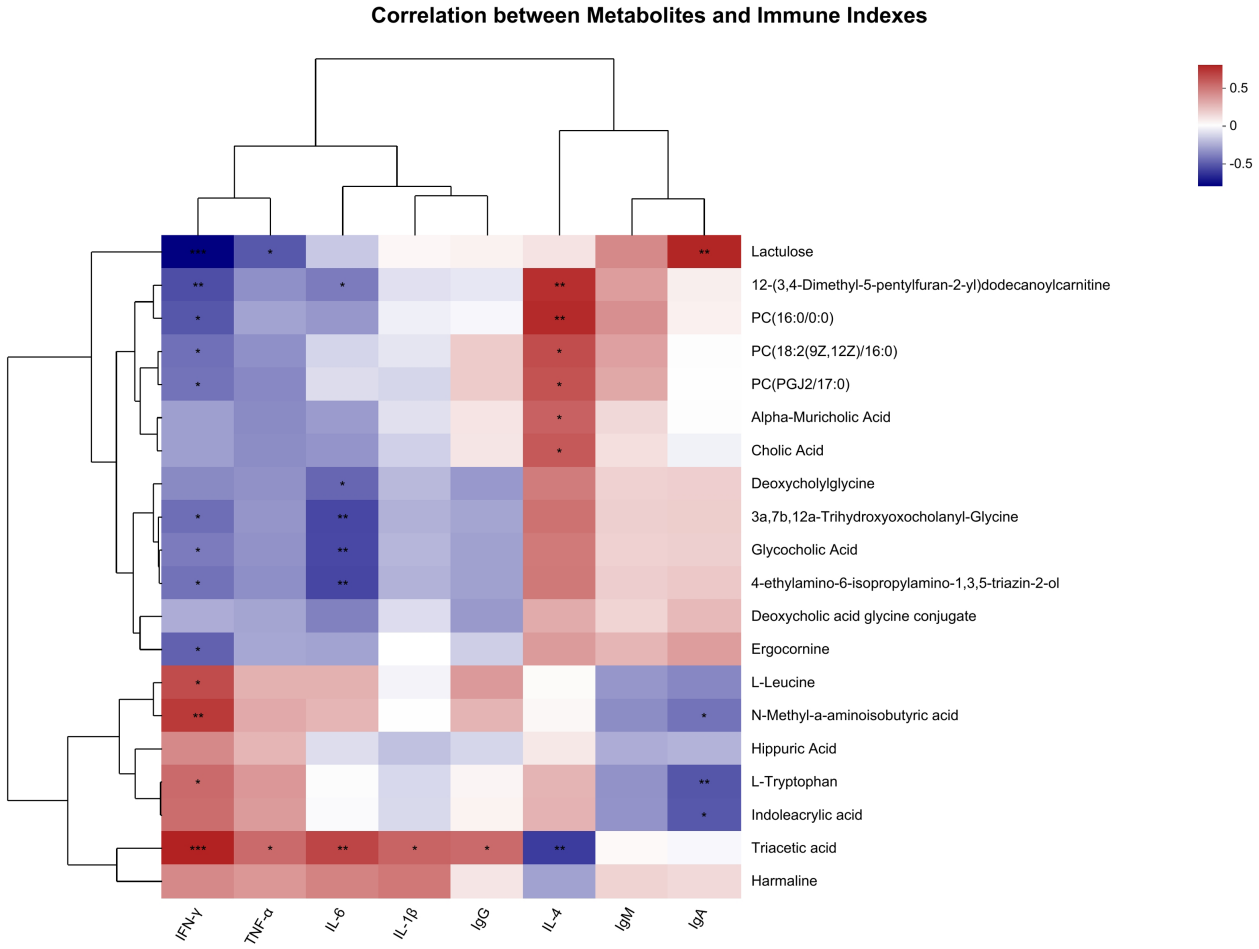
Correlation of metabolites with immune index (*n* = 8).

Metabolites with immune indicators were correlated. The top 20 abundance metabolites were selected, and the heatmap of their top 50 abundance correlation features is presented in Figure 9. The IFN-γ levels were compared with triacetic acid, L-leucine, N-methyl-a-aminoisobutyric acid, and L-tryptophan showed a significant positive correlation and compared with lactulose, ergocomine, glycocholic acid, 3a, 7b, 12a-trihydroxyoxocholanyl-glycine, 4-ethylamino-6-isopropylamino-1, 3,5-triazin-2-ol, 12-(3,4-dimethyl-5-pentylfuran-2-yl) dodecanoylcarnitine, PC (16:0/0:0), PC (18:2 (9Z, 12Z)/16:0), and PC (PGJ 2/17:0) showed a significant negative correlation. TNF-α levels were compared with triacetic acid showed a significant positive correlation and compared with lactulose showed a significant negative correlation. IL-6 levels were compared with triacetic acid showed a significant positive correlation and compared with deoxycholylglycine, glycocholic acid, glycocholic acid, 3a, 7b, 12a-trihydroxyoxocholanyl-glycine, 4-ethylamino-6-isopropylamino-1, 3,5-triazin-2-ol, 12-(3,4-dimethyl-5-pentylfuran-2-yl) dodecanoylcarnitine showed a significant negative correlation. IL-1β and IgG levels were compared with triacetic acid showed a significant positive correlation. IL-4 levels were compared with 12-(3,4-dimethyl-5-pentylfuran-2-yl) dodecanoylcarnitine, PC (16:0/0:0), alpha-muricholic acid, cholic acid, PC [18:2 (9Z, 12Z)/16:0], and PC (PGJ 2/17:0) showed a significant positive correlation and compared with triacetic acid showed a significant negative correlation. IgA levels were compared with lactulose showed a significant positive correlation and compared with N-methyl-a-aminoisobutyric acid, L-tryptophan, indoleacrylic acid showed a significant negative correlation.

## Discussion

### Changes in perinatal biochemical indicators

During the transition period, plasma Glu levels were significantly associated with the NEB status in ruminants (21). Glu is critical for promoting maximal milk synthesis (22). As a precursor of lactose synthesis, it is also the main energy donor of living cells and an intermediate product of metabolism (3). In this study, the concentration of Glu was the highest at ap7, which may be a large consumption of its own energy materials before and after calving, the feed intake cannot satisfy the nutritional needs of the body, and the body will increase the production of endogenous glucose to maintain glucose homeostasis (8, 23). Ins is a key regulator of maintaining glycemic balance and inhibiting triglyceride lipolysis in adipocytes. Ins has a key role in maintaining the balance of glucose metabolism, with Ins concentration decreases before and after calving (24). In this experiment, the increase in Ins concentration before and after delivery may disrupt the balance of glucose metabolism. This result may be due to the fact that 1 ap7 and pp are in negative energy equilibrium. Moreover, the cows had the potential to develop IR symptoms before and after calving.

Compared with normal condition cows, transition cows secrete various hormones acting against Ins and show greater insulin secretion capacity with greater peak insulin (25). In this study, HOMA-IR at ap7 was significantly higher than at ap14, pp7, and pp14. With fetal growth, insulin resistance significantly increased.

The detection of CHO level in the blood is important for the diagnosis of postpartum health of cows. CHO is mainly synthesized by the liver, and lipid metabolism is mainly completed in the liver. In healthy conditions, fat mobilization accelerates after calving, and cholesterol content will continue to rise. In this study, the CHO content gradually decreased from ap7 and reached the lowest value on the day of delivery and gradually increased later. Consistent with healthy conditions in which fat mobilization accelerates after calving, the cholesterol content will continue to rise. DHDL, as the main lipoprotein for transporting cholesterol, may be an indicator to test whether cows enter NEB (26). This study found that the concentrations of DHDL and DLDL decreased from ap14 to pp1, with the lowest concentration at pp1. It is possibly due to fetal growth and increased maternal demand for cholesterol at the end of pregnancy (27).

### Changes in perinatal immune indicators

Immunoglobulin has antibody activity, and its increased content can improve the immune capacity of the animal body. Cytokines are multifunctional protein molecules produced by immune cells and certain non-immune cells after stimulation, which play important roles in immune defense and inflammatory processes (28–30). Immunoglobulin and cytokines jointly participate in the immune process and enhance the body’s defense ability. Due to stress, such as pregnancy and deliver, the tissue metabolism of cows during the transition period changes, resulting in the neuroendocrine and immune status, which reduces the immunity (31).

Studies have found that IFN-γ is reduced in the breast before delivery, indicating that susceptibility to disease is related to immune cytokines. Cytokines have important roles in regulating fat mobilization. It can serve as a marker of lipid metabolism (32). The highest IFN-γ levels were tested in ap7, consistent with the findings of Sordillo. This provides further evidence that the transitional disease susceptibility is known through immune cytokines. IgA prevents bacteria or viruses on the mucosal surface and attaches to the mucosa for antibacterial and antiviral protective effects (33).

IL-4 is an anti-inflammatory factor, and the highest level of IL-4 is found in pp7, which may be related to the occurrence of deliver stress. TNF-α has tumor cell-killing and immunomodulatory functions. It can stimulate the synthesis of IL-6, IL-8, and other interleukins, affecting lipid metabolism and sugar metabolism processes (34). IL-6 is dominated by humoral immunity and is a key component in inflammatory cytokines. Studies have shown that prenatal serum TNF-α and IL-6 in transitional cows can cause disturbance of immune function (35). The increase in TNF-α can make the body obtain a strong anti-inflammatory effect and accelerate the body’s response to inflammation of TNF-α, and IL-6 levels were minimized at pp7 probably because of the lack of carbohydrate intake after delivery, mobilizing large amount of fat for energy, affecting lipid metabolism and glucose metabolism processes, and causing immune dysfunction.

### Overall metabolic pathways mainly in amino acid metabolism and lipid metabolism

The physiological mechanisms associated with delivery in cows are still unclear. Most previous studies have focused on the metabolic regulation of cows by dietary additives, while the metabolism of ruminants itself before and after delivery is rare (36, 37). Therefore, our study analyzed the changes in the prenatal and postnatal blood metabolome. There were two main types of enriched metabolic metabolites: one is amino acid metabolism, the main metabolic pathways were tryptophan metabolism, tyrosine metabolism, and lysine degradation; the second is lipid metabolism, the main metabolic pathway was alpha-linolenic acid metabolism.

Amino acids are the final absorbed form of the protein. Catabolism of amino acids is also a necessary process to participate in energy production under the negative energy balance. Tryptophan is an important substrate for protein biosynthesis that reduces animal consumption and requires maintenance (38, 39). Tryptophan deficiency can hinder the accumulation of fat in the body. The catabolic pathway of tryptophan is catabolized through the canine urine pathway, promoting the production of antimicrobial peptides to alleviate intestinal inflammatory response and regulating intestinal immune tolerance (40, 41). Tyrosine can be used in the synthesis of adrenaline and thyroxine and regulate glucose metabolism and fat metabolism in the body. The results of this test showed that the tryptophan and tyrosine metabolic pathways were upregulated in prenatal cows than in postnatal metabolism, indicating that the breakdown of tryptophan and tyrosine is promoted, which is not conducive to lipid synthesis in cows. Lysine is one of the ketogenic amino acids when the lack of available carbohydrate can participate in the formation of ketone body and glucose metabolism. A precursor of the synthesis of botulinum alkali is involved in fat metabolism. The significant upregulation of lysine indicates that the ketogenic pathway is more active before delivery than after delivery. Most of glucose which needs in transtion period is provided by liver gluconeogenesis (42). Thus, glycogen coneogenesis is significantly upregulated before delivery to maintain the stable Glu levels (43).

Strengthening lipid metabolism can relieve the body in negative energy balance due to calving and lactation. Alpha-linolenic acid is an essential unsaturated fatty acid that constitutes the fat of the animal body. It is mainly the direct deposition of fatty acids obtained from the diet or the *de novo* synthesis of fatty acids in the body. The results of this test showed that alpha-linolenic acid was downregulated in postpartum cows compared with prenatal production, which may be related to the involvement of fatty acids in lipid synthesis in blood.

### Correlation mainly concentrated in lipid and lipid-like molecules, organic heterocyclic compounds, organic acids, and their derivatives

To explore deeply, the top 20 metabolites were associated with glucose metabolism indicators, lipid metabolism indicators, and immune indicators and found that the metabolites were mainly concentrated in lipid and lipid-like molecules, organic heterocyclic compounds, organic acids, and their derivatives. Moreover, these metabolites were upregulated relative to the prenatal period. Bile acids and their derivatives were negatively correlated with glucose metabolism indicators and positively correlated with lipid metabolism indicators. Bile acids come from the catabolism of cholesterol and are a physiological detergent that can promote the digestion and absorption of fat in the intestine and liver (44). DHDL can carry cholesterol in the tissue into bile acid (45). Deoxycholic acid glycine conjugate can dissolve fat for absorption and is absorbed. Cholic acid is the primary bile acid produced by the liver. Moreover, when the content is particularly high, it will damage the liver (46). According to the test results, fat mobilization was accelerated, insulin content and glucose content decreased, total cholesterol content increased, and fat metabolism was more vigorous after calving, possibly because bile acid promotes liver glycogen synthesis (47, 48). In addition, they can also regulate bile flow and lipid secretion and are involved in all key enzyme regulation of cholesterol homeostasis (49–51). According to the test results, bile acids and their derivatives were negatively correlated with IL-4 and were positively correlated with IL-6. Through its correlation, it can improve the body’s immune function and maintain its physical health (52–54).

L-tryptophan and indoleacrylic acid belong to indao and its derivatives, and these metabolites were downregulated relative to the prenatal period. The increased absorption capacity of sugars will increase the concentration of HbA1c, which will then affect the tryptophan absorption. Tryptophan is a precursor of serotonin. Serotonin plays a key role in regulating energy metabolism, locomotor activity, and dietary behavior. In turn, the effect of serotonin on metabolic processes is through the activation of the signaling pathway in hypothalamic neurons (55). The metabolism of tryptophan to serotonin requires nutrients such as vitamin B6 and niacin. Niacin is synthesized by canine urine and quinolinic acid. Two tryptophan anabolites were obtained by the canine urine pathway, and canine uric acid, indao, and its derivatives were negatively correlated with IgA and were positively correlated with IFN-γ. It is possible that indoleacrylic acid promotes intestinal epithelial barrier function, stimulates the production of butyryl acrylic acid, and subsequently alleviates the inflammatory response (56).

L-Leucine is a branched-chain amino acid that participates in energy and muscle metabolism. Leucine is only used in the metabolic pathway of ketogenic fat, and the metabolic end products are acetyl-coenzyme A and acetoacetate. Leucine, like other branched-chain amino acids, is implicated in insulin resistance (57). Postnatal L-leucine showed downregulation, positively associated with insulin resistance and IFN-γ, probably because leucine stimulates insulin release and promotes protein biosynthesis (58). This suggests that IFN-γ levels can indirectly indicate an association with the development of insulin resistance.

### Strengths and limitations of the study

Although there have been many studies on transitional metabolic disorders, there is still no clear and effective prevention and treatment options in production. Moreover, the results and effects of previous studies are more for other animals, which has great limitations for cows. Whether they are able to better prevent and ameliorate cow in the transition period metabolic disorders through synergistic interaction is not known. This experiment used the metabolic mechanism of cows in the transition period as the entry point and then reveal the different metabolic mechanism and mechanisms of blood metabolites and metabolic pathways at different times in the transition period. These results provided evidence for further exploration of the mechanisms of transitional metabolic abnormalities in dairy cows and could help in the development of new metabolic strategies.

Twenty transitional cows were randomly selected and eight healthy Holstein cows with similar body condition (3.25 ± 0.5, five-point scale for body condition score), weight (570 ± 50 kg), and similar pregnancy days were finally selected for testing. The sample size of eight cows is quite small. But it still laid a certain foundation for other investigators.

## Conclusion

In this study, the results of measuring biochemical indicators and immune indicators found that the tissue metabolism of dairy cows changed during the transition period due to stress, such as pregnancy and deliver. The body mobilized a large amount of fat energy, affecting the lipid metabolism and glucose metabolism processes. It potentially develops both IR and NEB, leading to immune dysfunction. Metabolic changes in transition cows were investigated using an untargeted LC-MS-based metabolomics approach. The global metabolic pathways identified by differential metabolites were mainly involved in amino acid metabolism, lipid metabolism, and carbohydrate metabolism; the key pathways with the highest correlation with metabolites include tryptophan metabolism, alpha-linolenic acid metabolism, tyrosine metabolism, and lysine degradation. These illustrated the metabolic changes in lipids and amino acids during the transition period. Lipid metabolism should be enhanced after delivery to relieve the NEB produced by the body. These data will provide a better understanding of the mechanisms of metabolic disorders during the transition. These may lay the stage for proposing new preventive and control strategies to regulate metabolic disorders, metabolic health, and immune function during the transition period.

## Epilogue

The experiment conducted the study of blood glucose metabolic indicators, serum metabolites and serum metabolic pathways at different times in the transition period and to explain the potential metabolic mechanism. In the next trial, we will increase the sample size and evaluate the key parameters of metabolic changes in lactation performance. Further determine the representativeness of the results of this study and strive to provide practical and effective prevention programs for production pastures.

## Data availability statement

The original contributions presented in the study are publicly available. This data can be found at: www.ebi.ac.uk/metabolights/MTBLS9144 (9, 59).

## Ethics statement

The experimental procedures were approved by College of Animal Science and Veterinary Medicine, Tianjin Agricultural University, Tianjin, China. The Animal Experiment Scheme (protocol number 2021LLSC27) has been audited by the Experimental Animal Ethics Committee, which conforms to the principles of animal protection, animal welfare and ethics, and conforms to the relevant provisions of the national experimental animal welfare ethics.

## Author contributions

XiZ: Writing – original draft, Data curation, Investigation, Methodology, Software. YW: Data curation, Project administration, Software, Validation, Writing – review & editing. LW: Conceptualization, Investigation, Validation, Writing – review & editing. SS: Formal analysis, Supervision, Validation, Writing – review & editing. CL: Resources, Validation, Visualization, Writing – review & editing. XuZ: Conceptualization, Methodology, Supervision, Validation, Writing – review & editing. LC: Formal analysis, Funding acquisition, Visualization, Writing – review & editing. YT: Funding acquisition, Methodology, Project administration, Writing – review & editing.

## Footnotes

^1^ http://www.hmdb.ca/

^2^ https://metlin.scripps.edu/

^3^ https://cloud.majorbio.com

^4^ https://www.kegg.jp/kegg/pathway.html

^5^ http://pathways.embl.de
