# Supplementary material for: Differences of serum glucose and lipid metabolism and immune parameters and blood metabolomics regarding the transition cows in the antepartum and postpartum period
Source: Front Vet Sci. 2024 Feb 2;11:1347585. doi: 10.3389/fvets.2024.1347585 (PMC10869552; doi:10.3389/fvets.2024.1347585)
Supplement: Supplementary file 1 [file Table_1.docx]

**SUPPLEMENTARY TABLE1 |** Mobile phase elution gradient

positive ion mode

| **Time （min）** | **Flow rate （mL/min）** | **A （%）** | **B （%）** |
| --- | --- | --- | --- |
| 0 | 0.4 | 100 | 0 |
| 3 | 0.4 | 80 | 20 |
| 4.5 | 0.4 | 65 | 35 |
| 5 | 0.4 | 0 | 100 |
| 6.3 | 0.4 | 0 | 100 |
| 6.4 | 0.4 | 100 | 0 |
| 8 | 0.4 | 100 | 0 |

negative ions mode

| **Time （min）** | **Flow rate （mL/min）** | **A （%）** | **B （%）** |
| --- | --- | --- | --- |
| 0 | 0.4 | 100 | 0 |
| 1.5 | 0.4 | 95 | 5 |
| 2 | 0.4 | 90 | 10 |
| 4.5 | 0.4 | 70 | 30 |
| 5 | 0.4 | 0 | 100 |
| 6.3 | 0.4 | 0 | 100 |
| 6.4 | 0.4 | 100 | 0 |
| 8 | 0.4 | 100 | 0 |

The mobile phase consisted of A (95% water +5% acetonitrile + 0.1% formic acid) and B (47.5% acetonitrile +47.5% isopropanol +5% water +0.1% formic acid).
