# Supplementary material for: Differences of serum glucose and lipid metabolism and immune parameters and blood metabolomics regarding the transition cows in the antepartum and postpartum period
Source: Front Vet Sci. 2024 Feb 2;11:1347585. doi: 10.3389/fvets.2024.1347585 (PMC10869552; doi:10.3389/fvets.2024.1347585)
Supplement: Supplementary file 2 [file Table_2.docx]

**SUPPLEMENTARY TABLE 2 |** Mass Spectrum Parameters

| **Description** | **Parameter** |
| --- | --- |
| Scan type（m/z） | 70-1050 |
| Sheath gas flow rate（arb） | 50 |
| Aux gas flow rate（arb） | 13 |
| Heater temp（℃） | 425 |
| Capillary temp（℃） | 325 |
| Spray voltage（+）（V） | 3500 |
| Spray voltage（-）（V） | -3500 |
| S-Lens RF Level | 50 |
| Normalized collision energy（eV） | 20,40,60 |
| Resolution（Full MS） | 60000 |
| Resolution（MS^2^） | 7500 |
